# Self-Specific Stimuli Interact Differently than Non-Self-Specific Stimuli with Eyes-Open Versus Eyes-Closed Spontaneous Activity in Auditory Cortex

**DOI:** 10.3389/fnhum.2013.00437

**Published:** 2013-07-29

**Authors:** Pengmin Qin, Simone Grimm, Niall W. Duncan, Giles Holland, Jia shen Guo, Yan Fan, Anne Weigand, Juergen Baudewig, Malek Bajbouj, Georg Northoff

**Affiliations:** ^1^Mind, Brain Imaging and Neuroethics Unit, University of Ottawa Institute of Mental Health Research (IMHR), Ottawa, ON, Canada; ^2^Cluster Languages of Emotion, Free University of Berlin, Berlin, Germany

**Keywords:** eyes-open, eyes-closed, resting state, self, spontaneous activity, intrinsic activity, rest-stimulus interaction, self-specific stimulus

## Abstract

Previous studies suggest that there may be a distinct relationship between spontaneous neural activity and subsequent or concurrent self-specific stimulus-induced activity. This study aims to test the impact of spontaneous activity as recorded in an eyes-open (EO) resting state as opposed to eyes-closed (EC) on self-specific versus non-self-specific auditory stimulus-induced activity in fMRI. In our first experiment we used self-specific stimuli comprised of the subject’s own name and non-self-specific stimuli comprised of a friend’s name and an unknown name, presented during EO versus EC baselines in a 3 name condition × 2 baseline design. In Experiment 2 we directly measured spontaneous activity in the absence of stimuli during EO versus EC to confirm a modulatory effect of the two baseline conditions in the regions found to show an interaction effect in Experiment 1. Spontaneous activity during EO was significantly higher than during EC in bilateral auditory cortex and non-self-specific names yielded stronger signal changes relative to EO baseline than to EC. In contrast, there was no difference in response to self-specific names relative to EO baseline than to EC despite the difference between spontaneous activity levels. These results support an impact of spontaneous activity on stimulus-induced activity, moreover an impact that depends on the high-level stimulus characteristic of self-specificity.

## Introduction

Spontaneous (or intrinsic) neural activity is operationally defined as activity that is relatively stable during the so-called “resting state” in which a subject is physically and mentally at rest (but awake) and exposed to a minimized stimulus array. The potentially important role of the brain’s spontaneous activity has been suggested by findings that show such activity in many brain regions by a variety of methodologies (Panksepp, 1998; Raichle et al., 2001; Llinas et al., 2002; Freeman et al., 2003; Shulman et al., 2003, 2007, 2009; Raichle, 2009, 2010; Lauritzen et al., 2012). A question that suggests itself is what this role may be, including its contribution to or impact on the brain’s response to stimuli (Northoff et al., 2010).

One approach to this question that has emerged recently is based upon the finding that there is a strong overlap between regions that show high spontaneous activity in the resting state and those that show a response to self-specific stimuli and tasks, with this overlap particularly marked in cortical midline regions (D’Argembeau et al., 2005; Beer, 2007; Schneider et al., 2008; Qin and Northoff, 2011; Whitfield-Gabrieli et al., 2011). In contrast, responses to non-self-specific stimuli do not show such an overlap (Qin and Northoff, 2011). The overlap is suggestive of some form of distinct relationship between spontaneous activity and self-specific stimulus processing, possibly including an interaction between spontaneous activity and self-specific stimuli that is different than for non-self-specific stimuli. Such possibilities remain hypothetical but attractive and open to further investigation.

Preliminary work investigating the relationship between spontaneous and stimulus-induced activity in general has been carried out. For example, recent human imaging studies have shown that higher spontaneous activity immediately preceding a stimulus is predictive of higher stimulus-induced activity in the auditory cortex (Sadaghiani et al., 2009; Northoff et al., 2010). Similar effects have also been observed in visual cortex (Hesselmann et al., 2008) and somatosensory cortex (Boly et al., 2007).

An alternative approach was taken by Lerner et al. (2009), which attempted to modulate the level of spontaneous activity by using eyes-open (EO) and eyes-closed (EC) baseline conditions whilst stimuli consisting of musical tones were presented. It was found that the tones induced greater BOLD signal response in the auditory cortex during the EO than the EC condition. That said, a limitation of this particular study was that the spontaneous activity level itself in the auditory cortex during EO and EC conditions was not measured in the absence of stimuli (i.e., in the resting state). This makes it more difficult to interpret the observed effect as being a result of modulating spontaneous activity.

Modulation of spontaneous activity by EO versus EC can be seen in light of a growing literature on differences in brain activity produced by switching between these two states (Fox et al., 2005; Fransson, 2005; Barry et al., 2007; Yang et al., 2007; McAvoy et al., 2008; Bianciardi et al., 2009; Yan et al., 2009; Fingelkurts and Fingelkurts, 2010; Wu et al., 2010; Donahue et al., 2012). For example, in EEG, the mean power of the delta, theta, alpha, and beta bands is less in EO than EC across the scalp (Barry et al., 2007, 2011; Chen et al., 2008). In fMRI, functional connectivity between brain regions is weaker in EO than EC (Wu et al., 2010). Visual and auditory cortices show higher neural activity during EO than during EC (Marx et al., 2004; Qin et al., 2012a). Taken together, these studies demonstrate that EO versus EC can effectively change activity throughout large portions of the brain, including sensory and non-sensory regions.

Building on this described background, the aim of the current experiment was thus to investigate the question of the relationship between spontaneous and self-specific activity by presenting self-specific stimuli and non-self-specific stimuli during EC and EO using fMRI. In addition, we aimed to measure simple spontaneous activity (in the absence of stimuli) in the regions identified as being of interest in the main interaction [(self-specific, non-self-specific stimuli) × (EC, EO)] analysis. We used auditory stimuli for several reasons. Firstly, a robust differential response to auditory self-specific stimuli subject’s own name (SON) versus non-self-specific stimuli (other names) has been found in auditory cortex (Di et al., 2007; Qin et al., 2010). Correlations between spontaneous activity and stimulus-induced activity have also been seen in the same region (Sadaghiani et al., 2009; Northoff et al., 2010). Thirdly and from a practical perspective, the use of auditory as opposed to visual stimuli allowed for even-handed stimulus presentation during both EO and EC.

Our study is comprised of two experiments. The first of these is an investigation of the impact of the EO/EC dimension of spontaneous activity on self-specific versus non-self-specific auditory stimulus-induced activity in auditory cortex using EO versus EC baselines during stimulus presentation. We used self-specific versus non-self-specific stimuli in the form of the SON versus other names. Given that the overlapping between high spontaneous and self-specific stimulus-induced activity may indicate a distinct relationship between each other, and the previous study indicated that the brain regions with high spontaneous brain activity were involved in the self-specific processing (Gusnard, 2005), we hypothesized that the spontaneous brain activity change (EO versus EC) would impact activity induced by self-specific stimuli differently than by non-self-specific stimuli. In Experiment 2 we directly measured spontaneous activity in the absence of stimuli (i.e., the resting state) during EO versus EC to confirm a modulatory effect of the two baseline conditions in the regions found to show an interaction effect in Experiment 1.

## Materials and Methods

### Subjects

Both Experiments 1 and 2 used the same 18 subjects (15 female, 3 male, age 20–34 years, mean age 27.1). The subjects did not suffer from any medical, neurological, or psychiatric disorders. All subjects had first names consisting of two syllables as part of the design of Experiment 1 (see below). Experiments 1 and 2 were run on different days (interval 8.5 ± 7.25 days, mean ± SD across subjects). Informed written consent was obtained from all subjects. The study was approved by the ethics committee of the Free University of Berlin.

### Design

#### Experiment 1. Interaction between EO versus EC baseline and self-specific versus non-self-specific stimulus-induced activity

In Experiment 1 we investigated the effect of EO versus EC spontaneous activity on self-specific versus non-self-specific auditory stimulus-induced activity. Based on an established paradigm (Qin et al., 2012b) we used three name conditions. The SON was the condition of interest (self-specific), whilst the name of a friend of the subject (FN) and a name unknown to the subject (UN) were used as control conditions (non-self-specific). Unknown names were names in common usage but that did not belong to anyone personally known to the respective subjects. Names were all first names with two syllables (including SON, as per subject inclusion criteria) and of the same gender as the subject. All name stimuli were spoken by the same male researcher who was not known to the subjects and were presented at 75 dB. Mean duration was 541 ± 96 ms (mean ± SD).

The experiment was a 3 name condition (SON, FN, UN) × 2 baseline (EO, EC) factorial design. For each subject there was one run of EO and one run of EC. In each run there were three blocks each of SON, FN, and UN for a total of nine name condition blocks. A block was comprised of 10 presentations of the relevant name, once every 2 s. This meant that each block was 20 s in length. Inter-block interval was 40 s. The order of the blocks was pseudo-randomized within the EO and EC runs. Ordering of the EO and EC runs was counterbalanced across subjects.

During the EO block, the subject was instructed to keep their EO and fixate on a white cross displayed on a black background on the in-scanner screen. During the EC run, the subject was instructed to close their eyes prior to the run starting. In both runs, subjects were instructed to relax and listen to the names as they were presented. In both Experiment 1 and 2, below, an in-scanner camera was used to monitor the subjects and ensure that they followed the EO/EC requirements.

#### Experiment 2. EO versus EC spontaneous activity

In Experiment 2 we measured spontaneous activity itself in EO versus EC resting states in the whole brain. There were five blocks each of EO and EC, presented alternately. The duration of EO blocks was four TR’s and the duration of EC blocks was five TR’s with TR = 8 s. EC blocks were longer than EO to allow the brain sufficient time to stabilize in the EC activity pattern. The start of an EC condition was indicated to the subjects by a single tone at 1000 Hz and 75 dB for 100 ms whilst the start of an EO condition was indicated by a double tone comprised of two single tones with an interval of 80 ms. Additionally, an open eye or closed eye icon was presented on screen in the scanner. The tones were extremely short relative to the length of the resting state blocks and the icons were small, simple, and static, so we judged the practical value of these instructional signs to outweigh any minor impact as stimuli on spontaneous activity. Subjects were instructed to relax with EO or closed according to the tone/icon prompts.

### Data acquisition and processing

Images were acquired on a Siemens 3.0T MAGNETOM TrioTim syngo MRI scanner at the Free University of Berlin. A 3D anatomical image was first acquired using a fast SPGR sequence (TR = 1.9 ms, TE = 2.25 ms, FOV = 256 mm × 256 mm, matrix = 256 × 256, slice thickness = 1 mm) for functional image registration and localization. Data for Experiment 1 were acquired using an EPI sequence (TR = 2 s, TE = 30 ms, ° = 90°, FOV = 192 mm × 192 mm, matrix = 64 × 64, slice thickness = 3 mm, gap = 0 mm). Each volume had 37 axial slices, covering the whole brain. Data for Experiment 2 were acquired using the same EPI sequence as Experiment 1 except TR = 8 s. For Experiment 2 a sparse sampling sequence was be used in order to reduce the effect of scanner noise on spontaneous brain activity (Gaab et al., 2007a,b, 2008).

Functional data were processed using the AFNI software package (Cox, 1996). Data underwent 2D and 3D head motion corrections, masking for removal of the skull, and spatial smoothing using a kernel of 6 mm full-width at half-maximum. Data were then converted to MNI space and resampled to 2 mm isotropic voxels.

### Analysis

#### Experiment 1 main part. Interaction between EO versus EC baseline and self-specific versus non-self-specific stimulus-induced activity

One subject was excluded due to excessive head motion (>3 mm). The data from Experiment 1 were submitted to deconvolution analysis using a general linear model (3dDeconvolve, AFNI) to obtain a whole-brain voxel-wise map of estimated linear coefficients for the three name conditions relative to the two baselines, for a total of six coefficient maps: SON during EO, SON during EC, FN during EO, FN during EC, UN during EO, and UN during EC. The 10 name presentations in each block were regarded as 1 entirety (BLOCK model in 3dDeconvolve) in the deconvolution analysis. The 40-s inter-block intervals gave enough room for the modeling.

Since all coefficients are relative to their respective baseline, they discount any trivial contribution to activity of baseline level itself, isolating the stimulus-induced change from baseline and thus the presumed stimulus-induced component of activity. The approach here was intended to reveal any non-trivial effect of baseline as a statistical factor on the stimulus-induced component itself.

The whole-brain voxel-wise maps of coefficients for the three name conditions relative to the two baselines were entered into a 3 × 2 ANOVA (3dANOVA, AFNI). Interaction regions were identified as those regions showing a name × baseline interaction effect at an FWE-corrected threshold of *p* < 0.05 based on clusters of 80 or more voxels with an uncorrected *p* < 0.005, with the group mean of the whole-brain mask used for FWE correction (AlphaSim, AFNI). These interaction regions were then taken as ROI’s for subsequent analysis.

Mean coefficients across voxels were calculated for each ROI. One-sample *t*-tests on these coefficients (two-tailed, *p* < 0.05) were done for each of SON, FN, and UN during EO and EC baselines to test for stimulus-induced signal changes relative to baseline. Paired *t*-tests were then done to test for differences in stimulus-induced signal between baselines. Bonferroni correction (*p* < 0.05) was applied across the ROI’s.

#### The additional exploratory part of Experiment 1: stimulus-induced activity in brain regions involved in self-specific processing

In addition to the above main analysis, an exploratory analysis of the effect of the different EO and EC baselines on self-related stimulus-induced activity in regions, other than the auditory cortex, that are involved in self-specific processing was carried out. To identify these regions, a whole-brain voxel-wise contrast of self-specific (SON) to non-self-specific (FN and UN) stimuli was made. Prior work has shown that the brain response to FN and UN is differentiable and so these two non-self conditions were included for completeness. In the exploratory analysis, FN and UN were grouped together as this work has also shown that SON-related activity is differentiable from both of these conditions which could work as the control conditions for self-specific stimuli and so they were taken as together representing non-self-specific stimuli (Qin et al., 2012b). Since FN and UN may make the signal twice, we take half of each into the contrast [SON −0.5 (FN + UN)] (3dANOVA3, AFNI). Those regions identified as being more active during self-specific stimulus presentation (using an FEW-corrected threshold of *p* < 0.05) were then taken as ROIs and analyzed in the same manner as the auditory cortex ROIs described above.

#### Experiment 2. EO versus EC spontaneous activity

One subject was excluded due to excessive head motion (>3 mm). The data from Experiment 2 were submitted to deconvolution analysis using a general linear model (3dDeconvolve, AFNI) to obtain a whole-brain voxel-wise map of estimated linear coefficients for the contrast [EO – EC]. Mean coefficients across voxels were calculated for the ROI’s from both parts of Experiment 1. One-sample *t*-tests (two-tailed) were done to test for differences in spontaneous activity between EO versus EC. Bonferroni correction (*p* < 0.05) was applied across the three ROI’s from Experiment 1 Main Part (bilateral auditory cortex and left inferior parietal lobule, name condition × baseline interaction effect), and independently across the five ROI’s from Experiment 1 Additional Exploratory Part [posterior cingulate cortex (PCC), right/left inferior frontal gyrus (r/lIFG), right anterior insula (rAI), left temporoparietal junction (lTPJ), self-specific versus non-self-specific stimulus-induced activity].

## Results

### Experiment 1 main part. Interaction between EO versus EC baseline and self-specific versus non-self-specific stimulus-induced activity

The bilateral auditory cortex and left parietal lobule emerged as regions showing a significant name (SON, FN, UN) × baseline (EO, EC) interaction effect (Table 1).

**Table 1:** **Experiment 1 regions of interest identified by interaction effect of name condition (SON, FN, UN) and baseline (EO, EC)**.

Brain regions	Coordinates (MNI)			*t*-Value (mean)	Volume (mm^3^)
	*x*	*y*	*z*		
Right auditory cortex	63	−28	23	8.78	744
Left auditory cortex	−63	−35	13	7.46	640
Left parietal lobule	−36	−56	52	8.08	1432

*Cluster size >= 80 voxels (2 mm isotropic), *p* < 0.05 FWE corrected*.

*The coordinates are the peak coordinates*.

In left auditory cortex, one-sample *t*-tests for each of SON, FN, and UN during EO and EC baselines to test for stimulus-induced signal changes relative to baseline found significant changes for all conditions in all ROI’s: SON during EO (*t* = 6.13, *p* < 0.001 Bonferroni correction), SON during EC (*t* = 8.45, *p* < 0.001 Bonferroni correction), FN during EO (*t* = 7.07, *p* < 0.001 Bonferroni correction), FN during EC (*t* = 3.25, *p* = 0.005 Bonferroni correction), UN during EO (*t* = 7.63, *p* < 0.001 Bonferroni correction), UN during EC (*t* = 3.20, *p* = 0.006 uncorrected, *p* = 0.018 Bonferroni correction).

**Figure 1:**
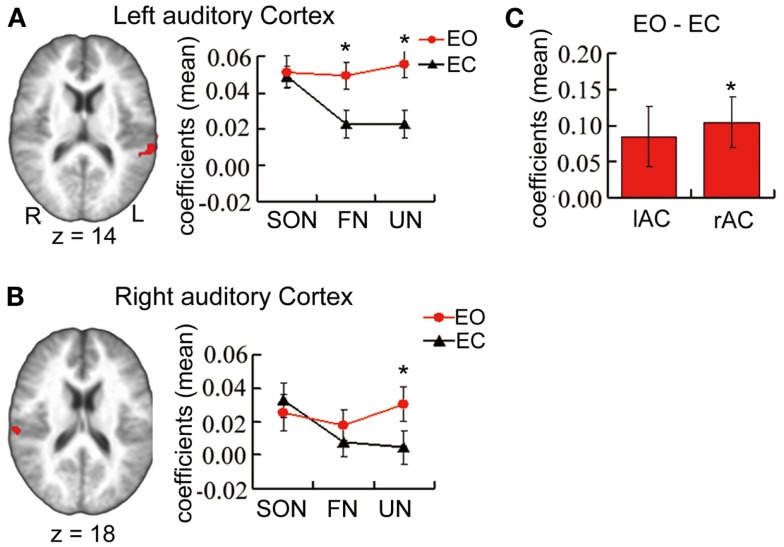
**(A,B)** From Experiment 1 main part. ROI’s showing a significant interaction effect in a 3 × 2 ANOVA of estimated coefficients for name condition (SON, FN, UN) and baseline (EO, EC) in bilateral auditory cortex. Graphs show estimated coefficients (mean across region ± SE) for name conditions relative to baselines. **(C)** From Experiment 2. Estimated coefficients of the contrast [EO – EC] in the same ROIs. r/lAC = right/left auditory cortex. *Significant difference.

Paired *t*-tests for differences in stimulus-induced signal between baselines revealed significantly stronger signal changes in UN (*t* = 3.95, *p* = 0.001 uncorrected, *p* = 0.003 Bonferroni correction) and FN (*t* = 3.51, *p* = 0.003 uncorrected, *p* = 0.009 Bonferroni correction) during EO than during EC. In contrast, no such difference was observed for SON (Figure 1A).

Results in right auditory cortex (Figure 1B) mirrored those in left. One-sample *t*-tests revealed marginally significant signal changes for SON during EO (*t* = 2.55, *p* = 0.022 uncorrected, *p* = 0.066 Bonferroni correction) and significant signal change for SON during EC (*t* = 3.40, *p* = 0.004 uncorrected, *p* = 0.012 Bonferroni correction), and for FN and UN during EO (*t* = 2.16, *p* = 0.046 uncorrected, *t* = 3.29, *p* = 0.005 uncorrected, *p* = 0.015 Bonferroni correction respectively) though not during EC.

Paired *t*-tests revealed significantly stronger signal changes for UN during EO when compared to EC (*t* = 3.47, *p* = 0.003 uncorrected, *p* = 0.008 Bonferroni correction). The difference for FN approached significance (*t* = 1.80, *p* = 0.09 uncorrected). No such difference was observed for SON.

In left inferior parietal lobule, one-sample *t*-tests showed that only SON during EO induced significant signal (*t* = 4.13, *p* = 0.001 uncorrected, *p* = 0.003 Bonferroni correction) while SON during EC did not. UN induced marginally significant signal changes during EO (*t* = 2.31, *p* = 0.035 uncorrected, *p* = 0.11 Bonferroni correction). Paired *t*-tests reveal that marginally stronger signal changes for SON during EO than EC (*t* = 2.592, *p* = 0.02 uncorrected, *p* = 0.06 Bonferroni correction). There is no difference for UN between during EC and EO while there is significantly stronger signal change for FN during EC than during EO (*t* = 2.829, *p* = 0.012 uncorrected, *p* = 0.036 Bonferroni correction).

### The additional exploratory part of Experiment 1: Stimulus-induced activity in brain regions involved in self-specific processing

**Figure 2:**
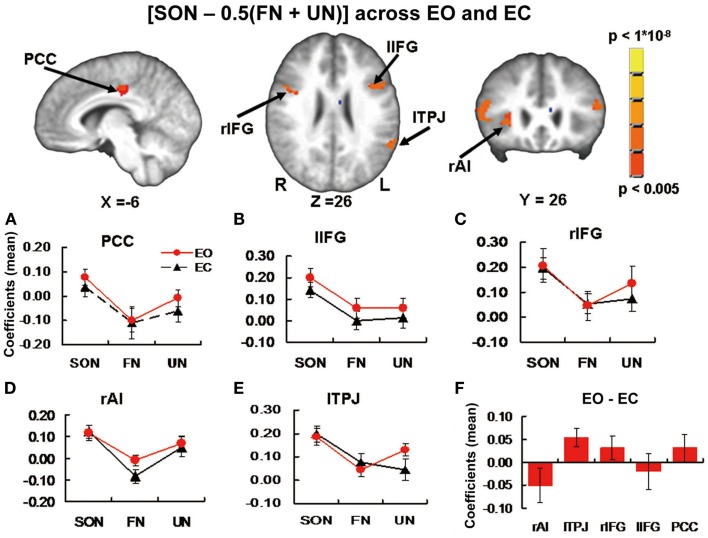
**Brain images and panels (A–E) from Experiment 1 additional exploratory part**. Activation Clusters in the contrast [SON −0.5 (FN + UN)] across EO and EC baselines. Panels show estimated coefficients (mean across region ± SE) for name conditions relative to baselines in each region. No significant differences were found when comparing stimulus-induced activity between baselines. **(F)** From Experiment 2. Estimated coefficients (mean across region ± SE) for the contrast [EO – EC] in the same ROI’s. Again no significant differences were found. PCC, posterior cingulate cortex; r/lIFG, right/left inferior frontal gyrus; rAI, right anterior insula; lTPJ, left temporoparietal junction.

To identify activation regions for the additional exploratory part of Experiment 1, the contrast [SON −0.5 (FN + UN)] across both EO and EC baselines yielded significant signal changes in five clusters in the PCC, bilateral inferior frontal gyrus (r/lIFG), rAI, and lTPJ respectively. Note that the cluster in rAI did not pass the FWE correction but we retained it since previous studies have shown this region to be involved in self-specific stimulus processing (Qin and Northoff, 2011; Qin et al., 2012b) (Table 2; Figure 2).

**Table 2:** **Experiment 1 (supplemental) regions of interest identified by activation for [SON −0.5 (FN + UN)] across EO and EC**.

Region	Coordinates (MNI)			Volume (mm^3^)	*t*-Value (mean)
	*x*	*y*	*z*		
Posterior cingulate cortex (PCC)	−6	−24	42	872	6.76
Right inferior frontal gyrus (rIFG)	50	11	11	2184	4.72
Left inferior frontal gyrus (lIFG)	−54	21	18	1928	6.28
Right anterior insula (rAI)	28	28	4	528	4.38*
Left temporoparietal junction (lTPJ)	−62	−50	21	1760	4.87

*Cluster size >= 80 voxels (2 mm isotropic), *p* < 0.05 FWE corrected*.

*Region did not pass FWE correction (see text).

*The coordinates are the peak coordinates*.

One-sample *t*-tests for each of SON, FN, and UN during EO and EC baselines to test for stimulus-induced signal changes relative to baseline revealed the following significant changes: in PCC, SON induced signal change during EO (*t* = 2.63, *p* = 0.018 uncorrected) and FN negative signal change during EO (*t* = 2.19, *p* = 0.043 uncorrected) (Figure 2A). In Left inferior frontal gyrus (lIFG), SON induced signal change during EO (*t* = 5.06, *p* < 0.001 Bonferroni correction) and EC (*t* = 4.09, *p* = 0.001 uncorrected, *p* = 0.005 Bonferroni correction) (Figure 2B). In Right inferior frontal gyrus (rIFG), SON induced signal change during EO (*t* = 3.33, *p* = 0.004 uncorrected, *p* = 0.02 Bonferroni correction) and EC (*t* = 5.03, *p* < 0.001 Bonferroni correction) and UN signal change during EO (*t* = 2.19, *p* = 0.044 uncorrected) (Figure 2C). In rAI, SON induced signal change during EO (*t* = 3.38, *p* = 0.004 uncorrected, *p* = 0.02 Bonferroni correction) and EC (*t* = 4.08, *p* = 0.001 uncorrected, *p* = 0.005 Bonferroni correction) (Figure 2D). In lTPJ, SON induced signal change during EO (*t* = 5.39, *p* < 0.001 Bonferroni correction) and EC (*t* = 5.57, *p* < 0.001 Bonferroni correction), FN signal change during EC (*t* = 2.41, *p* = 0.029 uncorrected), and UN signal change during EO (*t* = 5.10, *p* < 0.001 Bonferroni correction) (Figure 2E). Paired *t*-tests for differences in stimulus-induced signal between baselines revealed no significant differences.

### Experiment 2. EO versus EC spontaneous activity

In the two ROIs in auditory cortices from Experiment 1 main part (bilateral auditory cortex, name condition × baseline interaction effect), one-sample *t*-tests for signal differences between EO/EC resting states revealed higher spontaneous activity during EO than EC in right auditory cortex (*t* = 2.91, *p* = 0.01 uncorrected, *p* = 0.03 Bonferroni corrected) and a trend toward a similar difference in left auditory cortex (*t* = 2.01, *p* = 0.06 uncorrected) (Figure 1C). In the left inferior parietal lobule, the spontaneous activity did not show any difference between during EO and during EC.

In the five ROI’s from Experiment 1 Additional Exploratory part (PCC, r/lIFG, rAI, lTPJ, self-specific versus non-self-specific stimulus-induced activity), one-sample *t*-tests revealed no significant difference between spontaneous activity during EO versus EC. In lTPJ, a trend toward higher activity during EC was seen (*t* = 2.58, *p* = 0.02 uncorrected) (Figure 2F).

## Discussion

We report an interaction study between the EO versus EC variance of spontaneous activity and self-specific versus non-self-specific auditory stimulus-induced activity in fMRI. Non-self-specific stimuli (friends’ names and unknown names) induced significantly stronger BOLD signal changes relative to respective baseline during EO versus EC baselines in auditory cortex. In contrast, self-specific stimuli (subjects’ own names) did not induce different signal changes between baselines. Thus, our results show an interaction effect of self-specific/non-self-specific stimuli and EO/EC baseline. These findings are consistent with a previous brain imaging study (Lerner et al., 2009) as well as EEG studies (Griskova-Bulanova et al., 2011a,b) that indicate EO versus EC baselines can affect neural response to auditory stimuli. Our results extend these findings by showing that EO versus EC interacts with self-specific stimuli differently than non-self-specific.

In the same regions, our second experiment confirmed that spontaneous brain activity as directly measured in the absence of stimuli (i.e., the resting state) is modulated (increased) by EO versus EC. This finding is also consistent with previous studies that indicate EO can arouse the entire cortex (Barry et al., 2007, 2009) and that EO is associated with stronger activation than EC across sensory cortices, not just visual cortex (Marx et al., 2003; Brandt, 2006; Wiesmann et al., 2006; Hufner et al., 2009; Qin et al., 2012a).

In additional exploratory work, we also investigated the effects of self-specific versus non-self-specific names across both baselines in the whole brain. This yielded significant activity differences in PCC, rAI, lIFG, rIFG, and lTPJ (Figure 2), generally consistent with previous studies (Kelley et al., 2002; Northoff and Bermpohl, 2004; Mitchell et al., 2005; Northoff et al., 2006; Platek et al., 2006; Uddin et al., 2007; Zhu et al., 2007; Yaoi et al., 2009; Qin and Northoff, 2011; Qin et al., 2012b).

Considering our results further, spontaneous brain activity during EO was significantly higher than during EC (Figure 1C) in auditory cortex, and non-self-specific names yielded stronger signal changes relative to EO baseline than to EC (Figures 1A,B). These combined findings are consistent with previous findings in auditory cortex where higher spontaneous activity immediately preceding a stimulus predicts higher stimulus-induced activity (Sadaghiani et al., 2009). In contrast to non-self-specific names, there was no difference in response to self-specific names relative to EO baseline than to EC, despite the difference between spontaneous levels themselves.

In light of the general trend of interaction between spontaneous activity and stimulus-induced activity (higher resting state activity, stronger stimulus-induced activity) (Bianciardi et al., 2009; Sadaghiani et al., 2009; Hesselmann et al., 2010; Northoff et al., 2010; Donahue et al., 2012), one interpretation of these interaction results could be framed in terms of modulation of stimulus-induced activity by underlying spontaneous activity. Previous studies have indicated that spontaneous activity may be associated or involved with self-specific processing (Gusnard, 2005), This theory is consistent with the fact that in the resting state in which spontaneous activity is particularly pronounced, external input and engagement is minimized, allowing a balance to shift more toward internal (neuro-intrinsic as well as interoceptive) input, which is in general more self-referential. See Northoff et al. (2006) for a survey and meta-analysis of pertinent research results. Thus, we might expect self-specific stimulus-induced activity to be impacted more in step with spontaneous activity by factors that affect the latter such as EO versus EC. Meanwhile, we might expect non-self-specific stimulus-induced activity to be impacted in a manner more dissociated with spontaneous activity. Our finding here of no difference in self-specific stimulus-induced activity relative to spontaneous baseline as opposed to a significant difference for non-self-specific stimuli is in keeping with this theory.

It could be argued that the differences in stimulus-induced activity during EO and EC observed in this study are the result of modulation of attention. However, previous cross-modal studies suggest that attending more to visual stimuli tends to inhibit response to auditory stimuli in auditory cortex (Laurienti et al., 2002; Mozolic et al., 2008). Our findings were the opposite: friend’s names and unknown names induced higher activity during EO than EC, making an explanation based on attention more problematic than one based on spontaneous activity.

Aside from the auditory cortex, the left inferior parietal lobule also showed a name condition × baseline interaction effect. This result needs to be treated with caution, however, as of the 6 name condition × baseline combinations only SON during EO induced a significant signal change in the region. Moreover, the signal changes for FN during EC were stronger as opposed to weaker than during EO, which was inconsistent with the trend of our findings in other regions and may be inconsistent with the previous studies mentioned above. Finally, unlike in auditory cortex there was no difference between EO and EC spontaneous activity levels. The interaction effect in the left inferior parietal lobule may thus merit more investigation in the future to clarify these issues.

As mentioned in the introduction, EO versus EC can cause changes in activity throughout the brain. Some of these changes may be meaningfully categorized as changes in spontaneous activity that can directly contribute to stimulus processing. But others may not be – for example, a greater propensity for mind wandering during EC (Yan et al., 2009). The line here is certainly blurry. Future work could use both neural and behavioral measures to further address the distinction between modulation of spontaneous activity as it directly contributes to stimulus processing and modulation of other cognitive processes that affect stimulus processing more indirectly.

There is another issue that should be mentioned. It may be argued that the EO resting condition should be more properly seen as an activation state (Barry et al., 2007; Logothetis et al., 2009). Nonetheless, numerous studies have used an EO resting state with apparently reasonable justification (Fox et al., 2005; Fransson, 2005; Barry et al., 2007; Yan et al., 2009), for example, when spontaneous activity is to be related to the responses to stimuli that are presented visually. In addition, it should be considered that the brain receives constant input during both the EO and EC condition (auditory, proprioceptive, etc.), and so a differentiation between the EO and EC as an activation state or not becomes less tenable.

In summary, spontaneous brain activity during the EO resting state was significantly higher than during EC in bilateral auditory cortex and non-self-specific names yielded stronger signal changes relative to EO baseline than to EC. This supports the idea that spontaneous activity can impact neural response and processing of stimuli. From this perspective, it may be one-sided to generally investigate response to stimuli solely by varying those stimuli. Rather, it may be fruitful to vary both stimuli and spontaneous activity or baseline. Moreover, our results show that modulation of spontaneous activity did not affect self-specific stimuli as it did non-self-specific, suggesting that an impact of spontaneous activity on stimulus processing is complex at least insofar as it can depend on the high-level stimulus characteristic of self-specificity.

## Conflict of Interest Statement

The authors declare that the research was conducted in the absence of any commercial or financial relationships that could be construed as a potential conflict of interest.
